# How teeth, tusks and horny pads evolved together in sea cows

**DOI:** 10.1098/rspb.2024.1154

**Published:** 2024-08-14

**Authors:** Nicholas D. Pyenson

**Affiliations:** ^1^ Department of Paleobiology, National Museum of Natural History, Smithsonian Institution, Washington, DC 20013-7012, USA

**Keywords:** nerves, teeth, tusks, pads, sea cows

Nearly three centuries ago, the shipwrecked crew of the *St. Peter* were tired, hungry and unprepared for a new species that greeted them: a giant herbivorous marine mammal. Georg Steller, the ship’s naturalist, carefully documented this sirenian species that quietly inhabited the shores of what would become known as Bering Island, identifying its basic anatomy, some of its life history and, befitting his crew’s dire situation at the far western end of the Aleutian Islands, how to kill and eat them [1]. The expedition leader, Vitus Bering, died on the island, but Steller and his notes returned to Russia, only to fall into obscurity for over a century. In the interim, fur traders used knowledge of Bering Island and its plentiful sea cows as a stepping stone for exploitation farther east, towards Russian America [2], consuming the only extant population of this species in the process. By 1768, less than three decades after its discovery, Steller’s sea cow (*Hydrodamalis gigas*) was hunted to extinction [1,2]. Beyond a shortlist of dubious soft tissue fragments, most of what we can now understand about this extinct species derives from studies of its bones [3].

Sea cows—extinct and extant alike, all belonging to the broader clade Sirenia—remain interesting to biologists today for a variety of reasons. Like whales, this group of marine mammals has terrestrial ancestors, but from an entirely different part of the mammalian family tree: sirenians belong to Afrotheria, a group of mammals whose living relatives include elephants, rock hyraxes, aardvarks, tenrecs, elephant shrews and others with African endemicity or origins [4]. Like whales, sirenians underwent an ecological transition to obligate aquatic life during the middle to late Eocene (approximately 47–34 million years ago), following a sequence of locomotory transformations that parallel early whales: sirenians evolved paddle-like forelimbs and lost weight-bearing hindlimbs, concurrent with tail-propelled movement underwater using a tail fluke [5]. Eocene-age sirenians have been uncovered from India to Africa to Europe, North America and the Caribbean, complicating simple biogeographical scenarios for this coastal-dwelling clade [6,7]. Consequently, each new fossil sirenian has the potential to clarify or confound our understanding of their evolutionary history [4].

Whereas whales were always carnivorous, herbivory has constrained sirenian evolution: sea cows never succeeded in adopting a pelagic lifestyle, and they remained tied to the coastal environments where their primary food—marine angiosperms, seagrass rhizomes or kelp—are abundant [6]. Thickened ribs ensure that sea cows carry a form of permanent ballast for keeping their body horizontal to feed, yet sirenians show their afrothere connections most clearly in their mouths: like elephants, sirenians harvest plant matter, manipulated carefully by a well muscled muzzle, coupled with either use of paired tusks (as with dugongs) or ever-replacing molars (as with manatees); but unlike elephants, sirenians have keratinized (or horny) pads in the front of their mouths for processing plant material. And Steller’s sea cow? *Hydrodamalis* belonged to a lineage of cold-water-adapted dugong relatives in the North Pacific that lost teeth entirely over several million years, and likely cropped kelp solely using a keratinized dental pad [8]. Again, our only evidence comes from Steller’s reporting and from subsequent observations by a few of his contemporaries [1,8].

How did this strange outcome for dentition and feeding evolve in this group? And what evidence do fossil sirenians preserve to explain these changes over the past 50 or so million years? In a recent study, Hautier *et al*. [9] provide a comprehensive hypothesis for tooth loss and the evolution of keratinized mouth structures in sirenians by drawing on data from ontogeny, morphology, the fossil record and genomics. This kind of integrative work sets the stage for understanding whether evolutionary patterns of keratinized feeding structures in sirenians share common developmental paths and environmental triggers with other mammals and more distantly related vertebrates that have undergone such dramatic morphological transformations.

First, Hautier *et al*. [9] surveyed morphological variation in all extant sirenian species: three species of manatees (*Trichechus manatus*, *Trichechus inunguis* and *Trichechus senegalensis*) and one species of dugong (*Dugong dugon*), including any available fetal manatee and dugong specimens. Using microcomputed tomography (microCT) scanning, they specifically focused on the lower jaws (i.e. mandible) to understand the relationships among the highly mineralized teeth, the alveoli that house them (and sometimes persist when teeth are lost evolutionarily or ontogenetically) and the branches of vessels inside bone that communicate blood and nerves to these structures. Hautier *et al*. [9] pursued a solid strategy of following the bony pathways of the nerves in the mandibles: starting with the primary mandibular canal and mental branch, they identified tiny canaliculi that would have otherwise been impossible to visualize without CT imaging. In all cases—adult and fetal specimens of both *Trichechus* and *Dugong*—Hautier *et al*. [9] resolved canaliculi that would have carried vessels in life to vascularize and enervate tooth roots and vestigial alveoli (i.e. much diminished toothless sockets), including vestigial anterior alveoli that underlie the keratinized dental pad. While teeth and keratinized dental pads change in size and complexity through ontogeny, fetal specimens were crucial to clarify the vestigial identity of tooth alveoli (versus potential tooth loss through ontogeny or post-mortem decay). Again, what about *Hydrodamalis*, which lacked teeth entirely? Hautier *et al*. [9] show that canaliculi extend throughout the dorsal surface of the mandible from the anterior portion underlying a keratinized pad to the vestigial tooth row. These data, however, do not resolve whether the entire dental system of Steller’s sea cow was replaced by canaliculi (and an anterior pad), or if canaliculi represent vestigial elements of the dental system. CT scans from fetal or juvenile *Hydrodamalis* material would help distinguish between these two possibilities.

Of course, extant taxa provide an incomplete picture of evolutionary history. By focusing on the inner structures of sirenian mandibles, Hautier *et al*. [9] had a remarkable advantage in their investigation: mandibles are durable and relatively well preserved in the fossil record, they generally fit inside CT scanners, and their diagnostic features (especially when they carry teeth) make them focal points for systematic studies, which means that many fossil taxa are erected using mandibles as type or referred material. Extending their investigation to available fossil sirenians, they documented how canaliculi vary relative to tooth rows that include more complex tooth morphologies because stem sirenians from the Eocene and Oligocene (e.g. *Prorastomus*, *Libysiren* and *Eosiren*) possess identifiable incisors, canines, premolars and molars. The vagaries of preservation and morphology may potentially obscure canaliculi in these fossil taxa, but Hautier *et al*. [9] were able to identify incipient canaliculi in *Prorastomus*, the oldest sirenian in their dataset. The specimens of *Eosiren libyca* and *Halitherium taulannense* that they examined showed clear canaliculi originating from the mandibular canal anteriorly towards putatively vestigial alveoli (i.e. corresponding in homologous position and morphology to surfaces that support a keratinized pad in extant sirenians), along with smaller canaliculi near their cheek teeth (i.e. premolars and molars).

Hautier *et al*. [9] also included fossil sirenians to illuminate changes within crown Sirenia. The fossil record of stem trichechids is sparse and the inclusion of *Ribodon limbatus* should reveal changes in manatee evolution, but the absence of teeth and poor preservation of the specimen Hautier *et al*. [9] examined mostly continue the enigma, despite canaliculi being abundantly visible in the posterior tooth row. *Rytiodus capgrandi*, a fossil dugongid in their dataset, mostly parallels the tooth and canaliculi morphology in extant *Dugong*. Although *Rytiodus* is an Early Miocene taxon, their finding is not particularly surprising given the gross similarities these two taxa share as dugongids. Future studies should examine the mandibular morphology of other dugongids, especially older Oligocene ones, which compose a species-rich stem group. Moreover, it will be interesting to know the results for hydrodamaline dugongids, which are abundant and well preserved in Neogene strata from North America [8]. It is likely that similar investigations of Miocene *Dusisiren* spp. and Pliocene *Hydrodamalis cuestae* will reveal the precise sequence of tooth loss and canaliculi development leading to *H. gigas*, especially because vestigial teeth are evident in juvenile specimens of *H. cuestae* [8].

Genomic datasets also provide another class of evidence to examine the evolution of tooth loss in sirenians. Using DNA from manatees, dugongs and Steller’s sea cow, Hautier *et al*. [9] investigated the functionality of dental genes that code for proteins responsible for enamel and dentine development in other mammals. As expected with their dentition, manatees show functional dental genes, but Hautier *et al*. [9] noted potential pseudogenization of *ACP4* in *Dugong* and *MMP20* in *Hydrodamalis*. More interestingly, Hautier *et al*. [9] found that the edentulous *Hydrodamalis* showed inactivated genes (*ACP4*, *AMBN*, *ENAM* and *ODAM*) related to enamel development, but found no evidence for pseudogenization of genes relative to dentine formation (*DSPP*) or tooth loss (*ODAPH*), implying that *Hydrodamalis* retained the genetic machinery to grow dentine, possibly covered by thin enamel. This hypothesis is consistent with the presence of cheek teeth in *Dusisiren*, and would be further supported by the discovery of canaliculi surrounding cheek tooth alveoli in other hydrodamaline dugongids and close relatives (e.g. *Metaxytherium arctodites*).

The main takeaway from the integrative work by Hautier *et al*. [9] is the primacy of neurovasculature for resolving seemingly disparate morphological states in the sirenian mouth through geological time. More fossil data from stem lineages (i.e. fossil taxa within crown Sirenia) would resolve the morphological transformations from ancestral states in stem sirenians to those observed in manatees, dugongs and StelIer’s sea cow. A broader fossil dataset would provide the basis to analyse the relative evolutionary rates of tooth loss and canaliculi development, while also accounting for trends in dental complexity (e.g. simplification, tusk enlargement and replacement systems) across co-occurring lineages in time and space [10]. In the case of Steller’s sea cow, both fossil data from extinct hydrodamalines and ontogenetic data from fetal or juvenile specimens would clarify the relationship between tooth loss and soft tissue evolution in this lineage.

The list of mammal groups that appear to use a common developmental mechanism to innovate structures concomitant with tooth loss (and simplification) includes anteaters [11], baleen whales [12] and sea cows now as well. More broadly, given the deep homology of dental genes that underlie dentition changes (e.g. [13]), it is likely that the co-option of canaliculi for innervating novel keratinized structures has been a feature of tetrapod evolution since at least the Mesozoic. Recently, Aguilar-Pedrayes *et al*. [14] showed that tooth row reduction in dinosaurs (including avian and non-avian lineages) was driven by the evolution of a rhamphotheca-like keratin beak, although they noted that the two did not evolve in a simple antagonistic relationship, despite developmental studies on beak development in bird embryos that suggest direct antagonistic interactions between tooth loss and rhamphotheca formation. Rather, Aguilar-Pedrayes *et al*. [14] showed that theropod dinosaurs independently reduced toothrows before evolving beaks, and that rostral keratin covers did not significantly increase the evolutionary rate of tooth loss.

Do these patterns have parallels in mammals? Such analyses of evolutionary rates of tooth loss and canaliculi development would be illuminating in fossil and extant sirenians, but lips and other orofacial morphology in sirenians [15] add complexity to the issue: dental pads have both upper and lower morphologies, and work in tandem with muscularized soft tissue to manipulate, crop and crush plant matter (dugongids also use tusks to varying degrees as part of the processing as well). Inferring orofacial soft tissues in fossil sirenians is an open opportunity to better integrate functional analyses of their feeding systems into an evolutionary framework. With more fossil and developmental data, future studies can build on Hautier *et al*. [9] by comparing the timing of feeding morphology changes in sirenians with body size changes [2,8,10] and their complex biogeographical history of dispersal and diversification [4–6] for a full picture of the evolutionary history of this group.

## Data Availability

This article has no additional data.
